# Exciting Times for Cytomegalovirus (CMV) Vaccine Development: Navigating the Pathways toward the Goal of Protecting Infants against Congenital CMV Infection

**DOI:** 10.3390/vaccines8030526

**Published:** 2020-09-14

**Authors:** Mark R. Schleiss, Don J. Diamond

**Affiliations:** 1 Department of Pediatrics, Medical School Campus, University of Minnesota, Minneapolis, MN 55455, USA; 2 Department of Hematology/HCT, Beckman Research Institute of the City of Hope, Duarte, CA 91010, USA

The congenital transmission of cytomegalovirus (cCMV) is the most common infectious cause of disability in children in the developed world, and probably globally [1,2]. Remarkably, in spite of the impact of cCMV on child health, overall awareness is low, particularly among women of child-bearing age [3]. Congenital infection can occur either in the context of primary maternal infection or re-infection of seropositive women with new strains of the virus that have polymorphisms in coding sequences in viral envelope glycoproteins (important targets of neutralizing antibody responses). The occurrence of such re-infections can be confirmed by the demonstration of the emergence of new antibody specificities in seropositive women [4]. Although the most severe disabilities associated with cCMV probably occur in the setting of a primary CMV infection that occurs in pregnancy (particularly early in pregnancy [5,6]), disabling congenital transmission can occur with a strain acquired during pregnancy through this re-infection phenomenon [7].

In light of these observations, the challenge of developing a vaccine to prevent congenital transmission seems formidable, since immunity conferred by immunization would need to be superior to that conferred by natural immunity. Moreover, in spite of the broadly acknowledged need for a vaccine, the lack of certainty about the key correlate (s) of protective immunity for the placenta and developing fetus further complicates vaccine development. Despite these challenges, the manuscripts in this special collection, “*Cytomegalovirus Infection and Vaccine Development*“, provide a fascinating snapshot of the emerging basic science, as well as the preclinical and clinical research observations that have helped point the way with respect to CMV vaccine research, and insights from these papers provide plenty of reasons to be optimistic!

A striking attribute of this collection of papers is the scientific diversity of the CMV vaccine-related topics presented. In the paper by Coppola and colleagues, the impact of maternal CMV antibody status on vertical transmission rates was studied using a novel mathematical approach, the Preferred Reporting Items for Systematic Review and Analysis (PRISMA) guidelines. The analysis confirms a low cCMV birth prevalence in highly seropositive populations, and estimates a placental transmission rate in non-primary infection lower than that of primary infection [8]. More data are needed, but this modeling provides support for the plausibility of preventing cCMV through vaccination. Molecular correlates of antiviral immunity at the placental level are explored in an elegant model of human trophoblast progenitor cells described by Tabata et al. [9]. These cells give rise to the mature cell types of the chorionic villi, cytotrophoblasts and multi-nucleated syncytiotrophoblasts. The fact that infection is blocked by monoclonal antibodies targeting CMV glycoprotein B as well as constituents of the CMV pentameric complex (PC) provides support for ongoing clinical trials of CMV vaccines. All CMV vaccines in clinical trials contain gB, either expressed alone or in combination with other CMV immune targets [10].

The interplay between gB, PC, and other CMV glycoproteins with their cellular ligands is reviewed in a state-of-the-art overview by Gerna et al. [11]. CMV cellular entry is complex, and exploits two distinct pathways that are cell-type specific and involve different cellular receptors. Interestingly, however, the gB protein is a key component of both the entry pathway into epithelial/endothelial cells, where interactions with receptors Nrp2 and OR14I1 with PC promotes the endocytosis of virus particles, followed by gB activation by the gH/gL/gO trimer-mediating virus entry via a low-pH pathway, and fibroblasts, where the interaction of the PDGFRα receptor with the gH/gL/gO trimer activates gB to mediate the fusion of the virus envelope with the cell membrane. Hence, there are clearly merits in including gB in the various CMV vaccine formulations currently undergoing clinical trial evaluation. Another paper in this Special Issue, published by Contreras and colleagues [12], compares modified vaccinia virus Ankara-vectored vaccines expressing gB as well as the PC in a preclinical congenital infection model in guinea pigs. Both vaccines improved pregnancy outcomes and reduced pup mortality compared to a vector-only control, but the gB vaccine resulted in a much more substantial reduction in maternal viral load (~700-fold) compared to the PC vaccine (5-fold).

The importance of gB as a vaccine target is further discussed in the paper by Gomes et al. [13], which reviews the efficacy against primary CMV infection (43% to 50%) identified in three independent phase II trials in immune competent subjects, as well as the studies in solid organ transplant patients that identified novel immunological and mechanistic correlates of protection, including antibody-mediated cellular phagocytosis (ADCP), engendered by the gB vaccine. The finding of ADCP was surprising, and this was noted both in these solid organ transplant studies [13,14] as well as in follow-up serologic studies of gB vaccinee sera from immunocompetent young adult studies [15,16]. Thus, this paper makes a case for the continuing need for mechanistic studies in CMV gB vaccinees, toward that evasive goal of ascertaining the elusive immunological correlates that provide protection against cCMV. Whether gB, with or without the PC proteins, will be sufficient for the induction of protective immunity in a cCMV vaccine remains unknown. In the paper by Gogesch et al. a strategy for the production of subviral dense bodies (DBs) is discussed [17]. DBs are synthesized in infected cell cultures and are released from these cells at late stages of CMV replication and are devoid of capsids and DNA (and are therefore non-infectious), but they contain the T-cell target pp65 (ppUL83) as well as viral envelope glycoproteins and hence may be a promising cCMV vaccine candidate. Clinical trials of this vaccine strategy are warranted.

Finally, this Special Issue of *Vaccines* offers two other papers that explore issues germane to CMV immunization that are often not considered in the discussion of the potential public health impact of a CMV vaccine—thus, these papers are “outside the box” with respect to cCMV, but deal with highly innovative concepts in CMV pathogenesis and vaccine biology, and not directly related to issues germane to congenital infection. The first paper, written by Wilski and Snyder [18], considers the interplay between CMV infection and cancer. This is relevant both to the concept of CMV-based therapeutic cancer vaccines, as well as to the potential causative role CMV may play in the pathogenesis and progression of human cancer. Human cancers with putative causal links to CMV infection include glioblastoma multiforme, colon cancer, and infant leukemia. Thus, the disease manifestations of CMV may extend far beyond those associated with congenital transmission or infections in transplant patients. In addition to cancer, CMV has also been implicated in other disease processes, ranging from atherosclerosis to immunosenescence. Although all of these associations remain unproven, a better clarification of the role that CMV may play in these diverse diseases can drive momentum to promote *universal* immunization against CMV, to capture indications other than the prevention of cCMV. Thus, it may be that the prevention of CMV infection confers benefits throughout the life course, and this should become an important component of the CMV vaccine dialog in the future. The second paper, from Ynga-Durand et al. focuses not on vaccines to prevent the acquisition of CMV, but on immunization using CMV itself as a vaccine vector, expressing heterologous antigens important in immune response to other pathogens [19]. These diseases include tuberculosis, HIV infection, *Listeria monocytogenes* infection, respiratory syncytial virus infection, herpes simplex virus infection, and malaria. This approach takes advantage of the fact that CMV reinfections occur in seropositive individuals; hence, CMV-seropositivity would not preclude the use of (for example) a CMV-vectored tuberculosis or an HIV vaccine for an individual at high risk of acquisition of either or both of these diseases. Key to the further development of this CMV-vectored vaccine strategy will be the engineering of the CMV genome in such a way as to render it apathogenic (such as through the targeted deletion of immune evasion/immune modulation genes), lest such a vaccine was to ever be inadvertently administered to a pregnant woman, while still maintaining robust immunogenicity.

In summary, these are exciting times for CMV vaccine development! Although maternal re-infections in seropositive women with subsequent vertical viral transmission continue to be a concern, the protective role of maternal preconception immunity is becoming better understood. A variety of vaccine platforms are in clinical trials, and the virus–host cell pathways of viral entry are being better elucidated, which in turn helps with rational vaccine design. Instructive small animal models of cCMV are available to help inform and direct vaccine strategies. An expanded role for CMV infection as a cause of human diseases over the life course [20], including cancer and immunosenescence, creates a framework whereby we might consider the eventual implementation of a universal vaccination program against CMV, toward a goal of eventual eradication. Finally, increased public awareness will help drive the social and political forces that motivate external funding agencies and pharmaceutical companies to dedicate resources toward the development of an effective vaccine. The collection of papers commissioned by Dr. Diamond in this issue of *Vaccines* covers the depth and breadth of these perspectives and provides a timely update on the hope that we may conquer this threat to child health and well-being.
